# Decoding multispecies oral biofilms: molecular architecture, host modulation and systems-guided precision anti-biofilm strategies

**DOI:** 10.3389/fcimb.2026.2002174

**Published:** 2026-10-29

**Authors:** Abdulrahman Abdullah Almusallam, Khaled S. Allemailem

**Affiliations:** Department of Medical Laboratories, College of Applied Medical Sciences, Qassim University, Buraydah, Saudi Arabia

**Keywords:** oral biofilm, oral microbiome, extracellular polymeric matrix, microbial coaggregation, functional genomics, multi-omics, spatial microbiology, precision anti-biofilm therapy

## Abstract

Oral biofilms are spatially ordered microbial communities whose pathogenic behavior is shaped by more than taxonomic composition. Their development reflects the combined effects of adhesin-receptor recognition, interspecies coaggregation, matrix formation, metabolite exchange, local physicochemical gradients and host-derived selection. Here, we synthesize molecular and ecological evidence on the bacterial cell surface-matrix interface and consider how these interactions contribute to cariogenic, periodontal and peri-implant biofilms. Particular attention is given to glucosyltransferase-dependent polysaccharides, extracellular DNA, amyloidogenic proteins, sortase-anchored surface factors and outer membrane vesicles, together with metabolic interactions involving lactate, arginine, hydrogen peroxide and heme. We further evaluate functional-genomic and systems-level approaches (including transposon sequencing, CRISPR interference, metagenomics, metatranscriptomics, metaproteomics, metabolomics, spatial imaging, genome-scale metabolic modeling and machine learning) while distinguishing association and prediction from experimentally demonstrated mechanism. Emerging interventions are assessed according to their biological target and evidence level, including adhesion blockade, specifically targeted antimicrobials, matrix synthesis inhibition and degradation, quorum interference, probiotics, catalytic nanoparticles, antimicrobial photodynamic therapy and antibiofilm dental materials. Across these approaches, reduction in biofilm biomass is not equivalent to ecological restoration or clinical benefit. Key translational barriers include model fidelity, donor-to-donor variation, spatial heterogeneity, treatment durability, endpoint selection and the limited external validation of many computational and preclinical findings. Future precision strategies should therefore integrate microbial identity, matrix architecture, spatial metabolism and host context, with experimental perturbation used to establish causal vulnerabilities before therapeutic translation.

## 1 Introduction: oral biofilms as spatially organized microbial ecosystems

The oral microbiome does not persist as a freely mixed suspension of microorganisms. On teeth, mucosal surfaces, the tongue and dental materials, microbial cells assemble into structured communities whose behavior emerges from interactions among neighboring organisms and from continuous exposure to host and environmental factors. Physical adhesion, metabolic exchange, intercellular signaling, competition for nutrients, extracellular matrix production and host-derived selection all contribute to this organization. Consequently, the biological properties of an oral biofilm cannot always be inferred from the behavior of its constituent species studied separately. Community composition matters, but so do the positions occupied by individual taxa, the molecules exchanged between them and the physicochemical conditions generated within the biofilm (Marsh and Zaura, 2017; Hajishengallis et al., 2023).

Imaging of human dental plaque has revealed reproducible micron-scale arrangements in which particular taxa occupy preferred positions relative to one another rather than being distributed randomly (Mark Welch et al., 2016). That positioning carries real functional weight: cells only a few micrometres apart can face markedly different nutrient concentrations, oxygen levels, pH and exposure to inhibitory metabolites. In cariogenic plaque, structured assemblies containing *Streptococcus mutans* can generate localized acidic zones at the tooth surface, linking microscale organization to an environment relevant to enamel demineralization (Kim et al., 2020). Where activity concentrates within the biofilm, in other words, is itself a product of this architecture.

Current models of caries have moved away from treating *S. mutans* as a sufficient explanation for disease. Its acidogenicity, acid tolerance, glucan production and role in cariogenic biofilm architecture remain mechanistically important, but these traits operate within a broader community. Cariogenic biofilms commonly contain several bacterial taxa and, in some settings, fungi, with interactions that shift between cooperation and competition. Repeated exposure to fermentable carbohydrates favors acid-producing, acid-tolerant functions, while the extracellular matrix modifies cell retention and solute transport. Disease therefore reflects both organism-specific activities and ecological conditions that favor a cariogenic consortium (Marsh and Zaura, 2017; Cai and Kim, 2023; Hajishengallis et al., 2023).

A comparable shift has occurred in periodontal microbiology. Current models place coordinated community activity within a susceptible and inflamed host environment rather than attributing periodontitis to the mere presence of a single pathogen. In the polymicrobial synergy and dysbiosis framework, organisms can occupy different functional roles, while community pathogenicity emerges through microbial cooperation coupled to a dysregulated inflammatory environment (Lamont and Hajishengallis, 2015; Hajishengallis and Lamont, 2021). Inflammation changes nutrient availability and physicochemical conditions in the periodontal pocket, while microbial activities influence the magnitude and quality of host responses. Dysbiosis and inflammation can therefore reinforce one another (Hajishengallis and Lamont, 2021).

Biofilm ecology is also relevant to implanted oral surfaces. Longitudinal analysis of early dental-implant biofilms showed complex, dynamic and strongly subject-specific communities; association analyses indicated numerous taxon-taxon relationships and a high potential for metabolic interactions (Dieckow et al., 2024). These observations caution against assuming that one standardized microbial composition adequately represents peri-implant biofilm development.

This raises a problem for therapies judged mainly by total biomass reduction or broad microbial killing. A lower cultivable count is evidence that something was killed, but on its own it says little about whether the functions driving disease were selectively suppressed, whether protective organisms survived, or whether the community stays altered once the selective pressure lifts. A treatment can also matter biologically even with only a modest effect on total biomass, provided it disrupts a key adhesion step, delays matrix maturation, interrupts a harmful metabolic interaction, or shifts the community toward a less damaging state (Marsh and Zaura, 2017; Cai and Kim, 2023; Hajishengallis et al., 2023).

None of this happens apart from the extracellular matrix. It provides binding sites, alters diffusion, retains enzymes and metabolites, and changes local mechanical and chemical conditions. These matrix functions arise at microbial surfaces, where adhesins, polysaccharides, pili, fimbriae, vesicles and extracellular nucleic acids mediate contact with neighboring cells and the host. For that reason, the cell surface and the matrix are more usefully considered as interacting parts of one functional continuum.

We organize this review around the sequence of molecular and ecological events that accompanies oral biofilm development. The discussion begins with attachment, receptor-mediated adhesion, coaggregation, intercellular signaling and spatial maturation, followed by the cell surface-matrix interface, metabolic heterogeneity and host modulation. We then examine functional-genomic, multi-omic, spatial and computational approaches that can move oral microbiome studies from association toward mechanism, and finally assess precision anti-biofilm strategies together with the translational obstacles that still limit durable disease control.

The distinctive aim of this review is not simply to catalogue oral biofilm mechanisms or therapeutic candidates. Instead, we connect reductionist molecular evidence to community-scale behavior through a common evidentiary sequence: association, mechanism, validation and translation. This allows cell-surface and extracellular-matrix biology, spatial metabolism, host feedback, functional genomics, multi-omics and computational prioritization to be considered within the same framework, while making clear where a conclusion is supported by direct perturbation and where it remains an inference from a simplified model.

Throughout the review, evidence is interpreted according to the biological context in which it was obtained. We use five working levels: Level A, monospecies molecular mechanism; Level B, defined pairwise or two-species interaction; Level C, defined multispecies community; Level D, patient-derived microcosm or ex vivo community; and Level E, *in situ* or human evidence. Levels A and B are indispensable for establishing mechanism, but they are treated as candidate determinants of complex-community behavior unless the same process is supported in Level C-E systems. This distinction is especially important for pathways first characterized in *S. mutans* or in highly controlled dual-species models.

In this review, we use the term “precision” in an operational sense rather than as a proxy for novelty. Specifically, precision can take several forms: organismal (selective targeting of a defined taxon), mechanistic (inhibiting a specific virulence factor, matrix component, or signaling pathway), spatial (preferential activity within a localized disease microenvironment), ecological (modulating pathogenic traits while sparing the surrounding commensal community), or host-centric (tailoring an intervention to a specific host or microbiome phenotype). Importantly, these modalities are distinct. A highly specific molecular inhibitor, for instance, can still trigger widespread ecological shifts, whereas a pH-responsive material may offer high spatial localization without any species-level selectivity.

### 1.1 Literature identification and evidence appraisal

Relevant literature was identified through PubMed/MEDLINE, supplemented by targeted searches of major publisher platforms and backward citation tracking from key primary papers and reviews. The search was last updated on 8 October 2026. Search terms were combined according to subsection topic and included oral and multispecies biofilms, extracellular matrix dynamics, coaggregation and coadhesion, spatial architecture, functional genomics (including *Tn-seq* and CRISPR interference), multi-omics approaches (metagenomics, metatranscriptomics, metaproteomics and metabolomics), quorum sensing and quenching, cross-kingdom interactions with Candida, biofilm resilience, antimicrobial resistance, precision therapeutics, nanozymes, probiotics, photodynamic therapy, peri-implant microbiology and microbiome machine learning.

Primary research articles were prioritized when evaluating specific mechanistic claims. Systematic and narrative reviews were cited primarily to provide broader clinical context and conceptual frameworks, while foundational landmark studies were retained where they established key mechanisms that remain central to the field today. As this is a narrative, conceptually structured review rather than a systematic review, no formal protocol registration or systematic risk-of-bias scoring was performed.

## 2 Molecular assembly of multispecies oral biofilms

Oral biofilm development is often summarized as a succession in which early colonizers attach, bridging organisms accumulate and later taxa join the community. That scheme is a convenient starting point, not a fixed schedule: individual organisms typically carry several adhesins at once, one adhesin can recognize more than one binding partner, and a cell’s physiology shifts once it makes contact or begins exchanging metabolites with a neighbor. What actually determines which cells stay together, and how those associations unfold, is a working network of host receptors, bacterial adhesins, receptor polysaccharides, signaling molecules, metabolites and matrix components.

For consistency, adhesion refers here to attachment of a microbial cell to a host, abiotic or matrix surface; coaggregation to specific recognition and aggregation between genetically distinct microbial cells; coadhesion to attachment of a planktonic cell to a microbial cell already attached to a surface; and aggregation to broader cell clustering without assuming a defined receptor-ligand mechanism. We likewise use pathogen for organisms with established disease-causing capacity, pathobiont for resident organisms whose disease-promoting activity is strongly context dependent, and opportunist when disease emerges primarily under altered host or ecological conditions. These labels are not treated as immutable species-wide properties.

In the oral cavity, enamel is rapidly covered by an acquired pellicle derived largely from salivary and gingival components (Marsh and Zaura, 2017), so arriving bacteria encounter adsorbed host proteins and glycoproteins rather than bare hydroxyapatite. *Streptococcus gordonii* shows just how specific this early recognition can be. Its antigen I/II-family proteins SspA and SspB, together with the serine-rich adhesin Hsa, mediate binding to immobilized salivary agglutinin/gp340, though the two adhesin systems differ in receptor preference and relative contribution (Jakubovics et al., 2005). This kind of redundancy may let pioneer colonizers stay attached even as pellicle composition varies across sites and individuals.

Direct examination of early human plaque found streptococci carrying the right receptor polysaccharides already arranged into mixed microcolonies with coaggregation-compatible bacteria during initial accumulation (Palmer et al., 2003). Actinomyces-streptococcal pairing offers a well-worked molecular example. Genetic dissection of *Actinomyces oris* type-2 fimbriae later pinned the coaggregation factor down to CafA, an LPXTG-containing tip protein: deleting *cafA* abolished coaggregation in the tested *A. oris*-*Streptococcus oralis* interaction, and both purified CafA and anti-CafA antibodies blocked it (Reardon-Robinson et al., 2014). These experiments refined the earlier view that assigned the coaggregation phenotype to the fimbrial structure as a whole.

Established bacterial cells subsequently provide receptors for additional colonizers. A well-characterized case is the *P. gingivalis*-*S. gordonii* interaction: the minor fimbrial protein Mfa1 recognizes a region of the *S. gordonii* antigen I/II-family protein SspB, and this contact is needed for efficient *P. gingivalis* biofilm development on the tested streptococcal substratum (Lamont et al., 2002). A surface molecule that first helped a pioneer colonizer engage host components, in other words, can go on to serve as a receptor for an entirely different bacterial species.

*Fusobacterium nucleatum* is routinely called a bridging organism because it associates with such a wide range of plaque taxa, but its binding is not one indiscriminate process. A genome-wide transposon screen confirmed that the outer-membrane adhesin RadD is central to interactions with several Gram-positive partners, and it tied that adhesion to metabolic regulation: the CarRS two-component system controls *radD* and lysine-metabolism genes, and free lysine inhibits RadD-dependent coaggregation (Wu et al., 2021). Partner binding therefore depends on more than adhesin availability alone: regulatory state and the surrounding metabolite environment also contribute to the interaction.

*Veillonella parvula* adds a further layer of partner specificity. Distinct trimeric autotransporter adhesins mediate attachment to streptococci and to *A. oris*, whereas coaggregation with *V. parvula* instead requires VisA on the *S. gordonii* side; disrupting either system reshapes mixed-biofilm architecture and species distribution (Dorison et al., 2024). Notably, coaggregation was not a prerequisite for every communication phenotype tested, a reminder that physical proximity, metabolic cooperation and signaling can overlap without being the same process.

Cell-cell signaling adds a regulatory dimension to these physical associations. Early genetic work in *S. mutans* linked the ComCDE-associated competence system to biofilm formation and architecture (Li et al., 2002), and the ComRS/XIP pathway offers a second route into competence. Sender-responder experiments showed that ComS/XIP-dependent signals pass between *S. mutans* cells, biofilms included (Kaspar et al., 2017), but the response depends heavily on context: mature biofilms need more signal to respond, and *comX* activation grows increasingly heterogeneous, with only part of the population reacting at all (Shields and Burne, 2016). A pathway mapped out in homogeneous planktonic culture, in other words, may not behave the same way inside a mature biofilm full of cells sitting in different physiological states.

Autoinducer-2 has its own role in oral interspecies communication. In saliva-fed dual-species communities, *Streptococcus oralis* and Actinomyces showed LuxS/AI-2-dependent mutualistic biofilm growth in a concentration-dependent manner (Rickard et al., 2006), and LuxS-dependent effects have likewise been reported in *S. gordonii*-*P. gingivalis* biofilms (McNab et al., 2003). LuxS phenotypes still deserve caution, though: LuxS feeds into the activated methyl cycle in addition to generating the AI-2 precursor, so a *luxS* deletion phenotype on its own does not prove a dedicated AI-2 quorum-sensing role.

This caveat matters especially for *F. nucleatum*. A 2026 study found detectable AI-2 production limited to subsp. *animalis* among the strains examined, with no experimental support for a conserved AI-2 quorum-sensing system across the *F. nucleatum* species complex (G C et al., 2026). Earlier statements about *F. nucleatum* AI-2 signaling made without specifying strain or subspecies should be read with that qualification in mind.

Taken across the whole community, these interactions add up to progressive spatial organization. Human plaque studies pick up tight streptococcal-actinomyces associations during early accumulation (Palmer et al., 2003), while mature communities settle into reproducible, taxon-specific architectures (Mark Welch et al., 2016); in cariogenic plaque, spatial arrangement links directly to localized acidic microenvironments (Kim et al., 2020), and deleting defined adhesins shifts species distribution within mixed biofilms (Dorison et al., 2024). “Early,” “bridging” and “late” colonizers remain convenient ecological labels, but plaque maturation is more accurately described as the progressive expansion of binding surfaces, metabolic niches and spatially constrained interactions, a sequence **Figure 1** summarizes.

**Figure 1:**
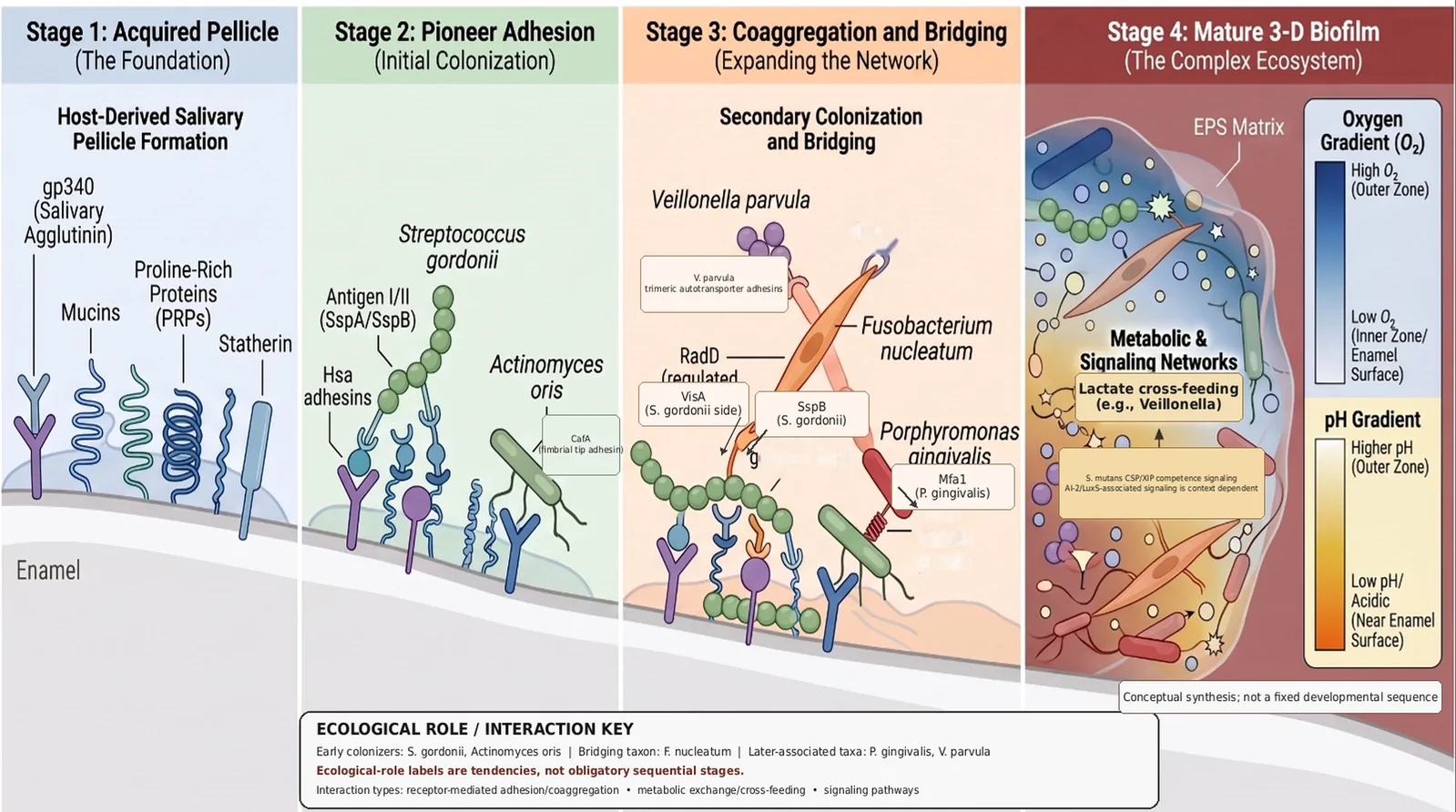
Assembly and spatial maturation of a multispecies oral biofilm. This image is an original graphical synthesis prepared for this review; the relationships it depicts are drawn from the cited primary literature. The image is a conceptual synthesis of the cited mechanistic literature rather than a reproduction of a single experimental dataset. Oral biofilm assembly begins on a host-derived acquired pellicle containing salivary receptors (Jakubovics et al., 2005). Pioneer organisms attach through defined adhesin-receptor interactions and subsequently provide bacterial surfaces for secondary coadhesion and coaggregation (Palmer et al., 2003). Representative molecular interactions include CafA-mediated Actinomyces-*Streptococcus* recognition (Reardon-Robinson et al., 2014), Mfa1-SspB interaction between *P. gingivalis* and *S. gordonii* (Lamont et al., 2002), RadD-dependent *F. nucleatum* coaggregation (Wu et al., 2021), and *V. parvula*-*S. gordonii* adhesin systems (Dorison et al., 2024). As the community matures, matrix production, signaling, metabolic cross-feeding and chemical gradients increase spatial heterogeneity (Mark Welch et al., 2016). Early, bridging and late colonizers are ecological tendencies here, not obligatory stages. The image presents these processes from left to right, with adhesion, metabolic exchange and signaling represented as distinct interaction types.

## 3 The extracellular matrix-cell surface interface

The extracellular matrix of an oral biofilm is not inert packing material surrounding otherwise self-contained cells. Its polymers are synthesized, released, retained and remodeled right at the microbial surface, while surface proteins and glycoconjugates govern how cells engage with them in the first place. A molecule that starts out as an adhesin can end up part of an extracellular assembly; an extracellular enzyme can build polymers directly onto a neighboring cell; DNA and membrane vesicles shed by one subpopulation can reshape the wider community. “Cell surface” and “matrix” are consequently working labels rather than a firm biological boundary, a continuum **Figure 2** summarizes.

**Figure 2:**
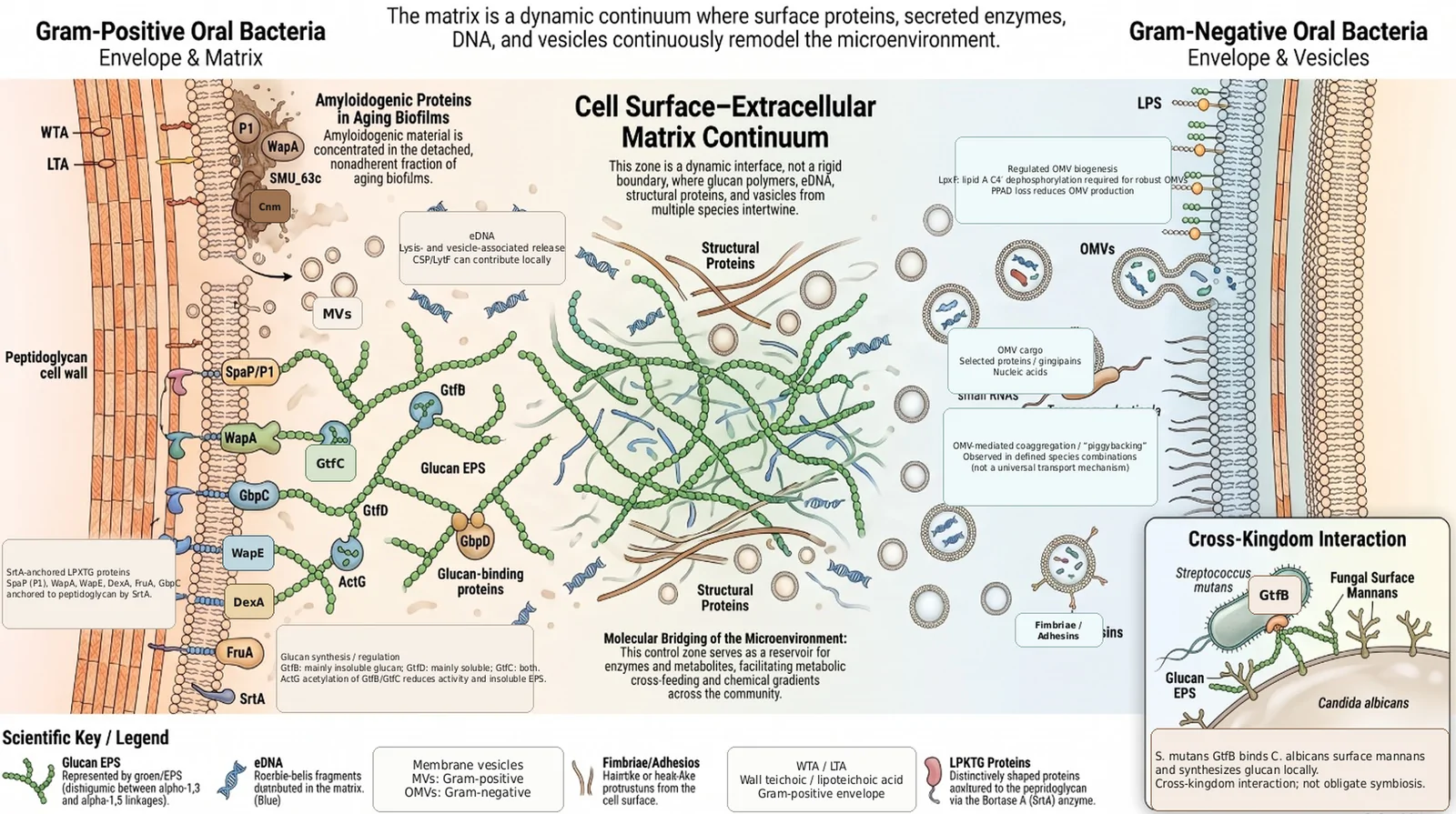
The extracellular matrix-cell surface continuum in oral biofilms. This image is an original graphical synthesis prepared for this review; the relationships it depicts are drawn from the cited primary literature. The image is a conceptual synthesis of the cited mechanistic literature rather than a reproduction of a single experimental dataset. The matrix takes shape through continuous interaction between cell-surface structures and extracellular components rather than arriving as an independent coating (Koo et al., 2010; Schormann et al., 2023). The Gram-positive panel depicts peptidoglycan-anchored LPXTG proteins, sortase A (Katsumata et al., 2022), glucosyltransferases, glucan-binding proteins (Lynch et al., 2007; Koo et al., 2010; Ma et al., 2021; Schormann et al., 2023; Huang et al., 2026), glucan EPS, eDNA (Liao et al., 2014; Nagasawa et al., 2020), membrane vesicles (Grenier, 2013; Ho et al., 2015; Wen et al., 2021) and stage-dependent amyloidogenic material (Besingi et al., 2017; Di Cologna et al., 2021; Yarmola et al., 2022). The Gram-negative panel depicts outer membrane architecture, adhesive structures, regulated OMV release and selected cargo (Vermilyea et al., 2021; Alaei et al., 2025), with a cross-kingdom inset showing *S. mutans* GtfB binding *C. albicans* mannans (Hwang et al., 2017). The two envelope panels are linked through a shared extracellular matrix, with amyloid explicitly identified as stage-dependent.

Sucrose-dependent glucan synthesis is probably the best-characterized matrix-building process in this system. In a multispecies model combining *S. mutans*, *S. oralis* and *Actinomyces naeslundii*, Gtf-derived exopolysaccharides generated localized binding sites and drove the formation of densely packed microcolonies (Koo et al., 2010). GtfB, GtfC and GtfD all share a GH70 glucansucrase fold but produce different glucans: GtfB leans toward water-insoluble glucan, GtfD toward soluble glucan, and GtfC toward a mix of both (Schormann et al., 2023). Losing GtfB or GtfC changes matrix accumulation and three-dimensional structure, which argues for complementary rather than fully redundant roles among the three enzymes (Koo et al., 2010).

Once synthesized, glucans interact with bacterial glucan-binding proteins. Mutational analysis of GbpA, GbpC and GbpD showed distinct effects on *S. mutans* biofilm depth and architecture (Lynch et al., 2007). Matrix synthesis and matrix recognition are therefore separate processes. Post-translational regulation adds another layer: GtfB and GtfC are substrates for ActG-mediated acetylation, and increased acetylation reduces enzymatic activity and water-insoluble EPS production (Ma et al., 2021). Recent microscale imaging further showed that sucrose-dependent EPS enhances stable early attachment and facilitates the transition from largely two-dimensional growth to three-dimensional architecture (Huang et al., 2026).

Extracellular Gtfs can also remodel cross-kingdom interactions. GtfB secreted by *S. mutans* binds the surface of *Candida albicans*, where it can synthesize glucan locally. Fungal outer-chain N-mannan and O-mannan contribute to this binding, and disruption of mannosylation reduces GtfB interaction and mixed-biofilm development (Hwang et al., 2017). The fungal surface can therefore serve as a platform for a bacterial extracellular enzyme.

Cross-kingdom effects extend beyond GtfB attachment itself. In a 2026 dual-species study, the S. mutans regulator GcrR altered C. albicans morphogenesis through the fungal Ras1-cAMP/PKA pathway while also changing bacterial glucosyltransferase and glucan-binding-protein expression, illustrating that bacterial-fungal coupling can operate through reciprocal regulatory effects rather than matrix binding alone (Zhang et al., 2026). Clinical and community-level studies also show that Candida, particularly *C. albicans*, is detected more frequently in plaque and saliva from children with early childhood caries than in caries-free children, although this association does not establish that fungal carriage is sufficient or causal for disease (O’Connell et al., 2020; Man et al., 2025).

eDNA is another structural constituent, but its origin is heterogeneous. *S. mutans* eDNA increases during biofilm growth and can arise through both lysis-associated and membrane-vesicle-associated pathways (Liao et al., 2014). DNase reduces biofilm formation, while DNA and GtfB-derived glucan can cooperate to enhance adherence (Liao et al., 2014). Competence-stimulating peptide can also induce heterogeneous LytF expression; a subset of LytF-expressing cells undergoes localized death and releases nucleic acids, particularly near the basal region of biofilms (Nagasawa et al., 2020). eDNA detected in mature plaque should therefore not be attributed automatically to nonspecific lysis.

*S. mutans* produces extracellular membrane vesicles containing proteins and DNA, and vesicle yield is influenced by multiple genetic determinants (Wen et al., 2021). Under sucrose-containing conditions, these vesicles can boost biofilm formation, consistent with a regulated contribution to matrix biology rather than mere membrane instability.

Proteins add yet another structural layer. P1, WapA and SMU_63c can each generate amyloidogenic extracellular material (Besingi et al., 2017), and Cnm forms amyloid too, losing its collagen-binding activity in the process (Di Cologna et al., 2021). It would be a mistake, though, to picture amyloid as a scaffold present from the earliest stages of the biofilm. Subsequent work found amyloid concentrated mainly in the nonadherent, detached fraction of aging *S. mutans* biofilms rather than in young or firmly adherent material, and P1 lost its adhesive function once amyloidogenesis occurred (Yarmola et al., 2022). Amyloid formation therefore looks temporally restricted, tracking changes that accompany an aging biofilm rather than defining it from the start.

Sortase-mediated anchoring, in Gram-positive bacteria, gives a direct molecular link between peptidoglycan and the extracellular world. A systematic survey identified six major SrtA-dependent proteins in *S. mutans*: SpaP, WapA, WapE, DexA, FruA and GbpC. Deleting *srtA* in *S. mutans* UA159 markedly reduced salivary binding, biofilm formation, coaggregation, surface hydrophobicity and binding to the extracellular-matrix proteins tested (Katsumata et al., 2022). These data provide a direct link between the cell envelope and oral biofilm architecture without implying that peptidoglycan-synthesis proteins in general are matrix determinants.

The surface-matrix interface is organized differently in Gram-negative periodontal organisms. *P. gingivalis* sheds large numbers of outer membrane vesicles (OMVs) loaded with selected lipids, proteins, nucleic acids and enzymatic cargo, and losing PPAD cuts OMV formation, reduces gingipain-associated proteolysis and changes surface behavior (Vermilyea et al., 2021). Lipid A remodeling also contributes: C4′ dephosphorylation by LpxF is required for robust OMV production in the tested *P. gingivalis* strain (Alaei et al., 2025). Released vesicles can transfer genetic material and modify *S. gordonii* biofilm maintenance (Ho et al., 2015); in other species combinations they also mediate coaggregation and “piggybacking,” as reported for *Treponema denticola* and *Lachnoanaerobaculum saburreum* (Grenier, 2013).

The matrix-cell surface continuum, then, is dynamic and varies by taxon rather than following one universal pattern. Gtfs build glucans that serve simultaneously as structural polymers and new binding substrates; Gbps link cells to those glucans; eDNA is released through both regulated and unregulated routes; vesicles carry DNA and proteins outward; and certain adhesins can convert into extracellular amyloidogenic material. If one component is disrupted, other processes such as adhesion, vesicle production, autolysis or matrix composition may also change. Matrix-directed studies should therefore assess architecture and cellular physiology alongside total biomass. **Table 1** summarizes representative molecular determinants of biofilm assembly and matrix biology discussed here and in the preceding section.

**Table 1:** Representative molecular determinants of oral biofilm assembly and maturation.

Organism/system	Molecular determinant	Biofilm process	Experimentally supported function	Evidence/model and context level	Ref.
*S. gordonii*	SspA/SspB, Hsa	Pellicle/host adhesion	Recognition of salivary host receptors including gp340 in tested systems	Mutant/receptor-binding experiments; A - monospecies/host-receptor assay	(Jakubovics et al., 2005)
*A. oris*	CafA on type-2 fimbriae	Interspecies coaggregation	Direct coaggregation factor for interaction with *S. oralis*	Deletion, purified protein and blocking antibody; B - defined pairwise interaction	(Reardon-Robinson et al., 2014)
*P. gingivalis*-*S. gordonii*	Mfa1-SspB	Secondary colonization	Required for efficient *P. gingivalis* biofilm development on tested streptococcal substratum	Genetic and biofilm assays; B - defined two-species substratum model	(Lamont et al., 2002)
*F. nucleatum*	RadD, CarRS	Coaggregation	RadD-dependent interaction regulated by CarRS and influenced by lysine	Genome-wide genetics plus biochemical validation; B - defined partner interactions	(Wu et al., 2021)
*V. parvula*-*S. gordonii*	Trimeric autotransporters; VisA	Coaggregation/spatial organization	Defined adhesins alter mixed-biofilm spatial structure	Genetic mixed-biofilm study; B - defined pairwise biofilm	(Dorison et al., 2024)
*S. mutans*	ComCDE/ComRS/XIP	Competence-associated signaling	Regulates competence and heterogeneous population responses	Mutant, reporter and sender-responder experiments; A - monospecies signaling	(Li et al., 2002; Shields and Burne, 2016; Kaspar et al., 2017)
*S. mutans*	GtfB/GtfC/GtfD	EPS synthesis	Distinct glucan-product profiles contribute to matrix and microcolony formation	Mutants, structural/biochemical and multispecies studies; A-C - mechanism through defined multispecies validation	(Koo et al., 2010; Ma et al., 2021; Schormann et al., 2023; Huang et al., 2026)
*S. mutans*	GbpA/GbpC/GbpD	Matrix recognition	Glucan-binding proteins shape biofilm architecture	Targeted deletion mutants; A - monospecies	(Lynch et al., 2007)
*S. mutans*	eDNA/LytF	Matrix development	eDNA contributes to structure; localized LytF-associated death provides one release route	DNase, microscopy and genetic studies; A - monospecies	(Liao et al., 2014; Nagasawa et al., 2020)
*S. mutans*	P1/WapA/SMU_63c/Cnm	Protein/amyloid matrix	Amyloidogenic conversion is protein- and stage-dependent	Biochemical, mutant and spatial studies; A - predominantly monospecies	(Besingi et al., 2017; Di Cologna et al., 2021; Yarmola et al., 2022)
*S. mutans*	Sortase A and LPXTG proteins	Cell wall-environment interface	Surface anchoring affects salivary binding, coaggregation and biofilm formation	Systematic mutant analysis; A - monospecies	(Katsumata et al., 2022)
*P. gingivalis*	PPAD/LpxF-regulated OMVs	Vesicle-mediated effects	Defined envelope processes regulate robust OMV production and biofilm-associated behavior	Genetic/biochemical studies with selected interspecies assays; A-B	(Vermilyea et al., 2021; Alaei et al., 2025)

Evidence-context levels used in **Table 1**: A, monospecies molecular mechanism; B, defined pairwise/two-species interaction; C, defined multispecies community; D, patient-derived microcosm or ex vivo community; E, in situ/human evidence. Lower-context levels establish mechanism but are not treated automatically as proof of operation in complex human communities.

## 4 Community metabolism and microscale heterogeneity

Metabolism within an oral biofilm is strongly shaped by spatial position. Cells separated by only micrometres can exchange carbon sources, amino acids, heme and other metabolites, whereas cells from the same species may experience different oxygen tensions, pH values and metabolite concentrations depending on their location. A bulk measurement of whole plaque cannot resolve this microscale heterogeneity.

Lactate is a well-established example of cross-feeding in oral communities. Oral streptococci produce lactate during carbohydrate fermentation, whereas Veillonella species use organic acids, particularly lactate, as major carbon and energy sources. In a saliva-fed flow-cell system, *V. atypica* induced *S. gordonii amyB* expression only in nearby mixed microcolonies, indicating that the effective interspecies response was restricted to short distances under flow (Egland et al., 2004). A 2024 five-species biofilm model added a further wrinkle: glucose and lactate concentrations together shaped community composition, with high glucose plus high lactate driving rapid acidification and *S. mutans* outgrowth, while low glucose with abundant lactate instead favored *V. parvula* and kept the bulk environment less acidic (Sangha et al., 2024). Lactate is therefore both a fermentation product and a substrate for its neighbors at once, and which role dominates depends on the surrounding carbon supply.

Amino-acid metabolism creates additional connections. *S. gordonii* metabolizes arginine through the arginine deiminase system, generating ATP, ammonia and ornithine. The ArcD antiporter mediates arginine uptake and ornithine export. Deletion of *arcD* impairs arginine metabolism and ammonia production and reduces *F. nucleatum* accumulation in dual-species biofilms; exogenous ornithine restores fusobacterial accumulation (Sakanaka et al., 2015). A pathway that can raise environmental pH can thus also generate a metabolite that supports another organism.

Metabolic exchange is accompanied by chemical competition. Several health-associated streptococci produce hydrogen peroxide during aerobic metabolism. *S. sanguinis* and *S. gordonii* also release pyruvate, which can chemically detoxify H2O2 and protect nearby cells, so the same network generates both an inhibitory molecule and a molecule that attenuates its effect (Redanz et al., 2020). More recent work identified glycerol catabolism as an additional H2O2-generating route in *S. sanguinis*, with glycerol enhancing competition against *S. mutans* (Taylor et al., 2024). A high-H2O2-producing *Streptococcus oralis* subsp. tigurinus J22 isolate disrupted *S. mutans* organization, reduced enamel demineralization ex vivo and reduced caries development in a rodent model, emphasizing strain-level variation in competitive phenotype (Kim et al., 2022).

Biofilm pH is similarly local rather than uniform. Confocal pH ratiometry in in-situ-grown human dental biofilms detected differences approaching two pH units among regions within the same biofilm. After carbohydrate challenge, pH tended to be lower at the base than at the upper surface and lower downstream than upstream under saliva flow (Kristensen et al., 2023). A 2025 microscale study showed that arginine supplementation raised local pH and modified community and matrix properties but did not homogenize the biofilm; microscale variation persisted and in some settings increased (Assar et al., 2025).

Oxygen gradients are coupled to, but not interchangeable with, pH gradients. Microelectrode measurements showed rapid oxygen consumption near the surface of naturally grown dental biofilms and effectively anoxic deeper regions under the tested conditions (Von Ohle et al., 2010). New pH-O2 ratiometry indicates that low pH and low oxygen can co-occur, but their relationship is community- and condition-dependent; in complex salivary biofilms they correlated more clearly than in monospecies *S. mutans* biofilms (Lynge et al., 2026). Accordingly, neither variable should be used as a proxy for the other.

Below the gingival margin, the available nutrient pool is different. Heme is particularly important for *P. gingivalis*, which relies on exogenous acquisition. *S. gordonii* GAPDH binds heme, and the *P. gingivalis* HmuY protein can remove heme from the GAPDH-heme complex, providing a defined mechanism for interspecies acquisition (Ślęzak et al., 2020). More recent analysis of HmuS connects intracellular heme processing with heme homeostasis and biofilm formation (Cierpisz et al., 2026). Together, these studies trace subgingival heme use from extracellular capture to intracellular handling.

Not every metabolic exchange is cooperative. A recipient may benefit from a metabolite without returning a measurable advantage to the producer, and a compound that is inhibitory under one condition may be neutralized or become irrelevant under another. Terms such as cross-feeding, competition and mutualism are therefore most informative when supported by measured metabolite flux and fitness effects rather than by co-occurrence alone. Oral biofilms are chemically heterogeneous environments in which nutrient supply, diffusion, matrix architecture and spatial arrangement determine which metabolic interactions become consequential.

## 5 Host modulation of oral biofilm formation

Host-biofilm interactions are reciprocal from the outset. Saliva, mucins, antibodies, antimicrobial peptides, gingival crevicular fluid and immune effectors shape attachment, nutrient availability and local chemistry; the biofilm, in turn, releases metabolites, proteases, vesicles and other products that alter epithelial and immune signaling. In periodontal sites this feedback can become self-reinforcing, because inflammation changes the nutrient and redox environment in ways that favor organisms adapted to an inflamed subgingival niche.

Host-derived conditions are present throughout oral biofilm development. Saliva coats surfaces, buffers local chemistry, supplies nutrients, carries antibodies and antimicrobial proteins, and redistributes microbial cells. Gingival and mucosal epithelia provide both a physical barrier and a signaling surface, while neutrophils, complement and other immune components become increasingly important near the gingival sulcus and periodontal pocket. These effects are not limited to microbial killing. Host factors can alter bacterial adhesion or gene expression, serve as metabolic substrates, and, once inflammation sets in, reshape the habitat in ways that favor organisms adapted to inflamed tissue.

MUC5B is a good illustration of this multifunctionality. Purified salivary MUC5B and the O-glycans it releases suppress *S. mutans* competence-associated signaling and cut genetic transformation, all without measurably slowing growth (Werlang et al., 2021). A host glycoprotein, in other words, can rein in bacterial phenotype without acting as a conventional antimicrobial. Health-associated organisms turn the same molecule to their own advantage, though: *S. gordonii* and *A. naeslundii* draw nutrients from MUC5B and show complementary metabolism during early biofilm growth (Robertsson et al., 2023). A 2025 study went further, showing that MUC5B shifted early attachment, species distribution and glucose-associated metabolism in ways consistent with a role in maintaining ecological balance, though it stopped short of demonstrating actual clinical restoration of eubiosis (Robertsson et al., 2025).

Secretory IgA offers a second non-bactericidal route of control. Human sIgA raised against antigen I/II inhibits *S. mutans* binding to saliva-coated hydroxyapatite (Hajishengallis et al., 1992). Natural plaque is certainly more complicated than this experimental system, but the result still establishes that adaptive mucosal immunity can shape community assembly by blocking attachment rather than by killing the organism outright.

Salivary antimicrobial peptides also show organism-specific activity. Histatin-5 protects oral tissue against *Candida albicans* in an ex vivo infection model (Peters et al., 2010), but a 2023 analysis found no comparable killing of seven tested oral or oropharyngeal streptococci at physiologically relevant concentrations (Stewart et al., 2023). It is therefore inaccurate to describe histatin-5 simply as a broad-spectrum oral antimicrobial peptide.

Gingival epithelial cells detect microbial components, secrete chemokines and cytokines, and produce beta-defensins. *P. gingivalis* exposure can induce hBD-2 and IL-8 in primary gingival epithelial cells (Taguchi and Imai, 2006), yet clinical tissue data show that defensin abundance does not simply rise in parallel with inflammation or bacterial burden (Özdemir et al., 2020). Multispecies composition further changes epithelial output: combinations of *Corynebacterium durum*, *S. sanguinis* and *P. gingivalis* produced responses not predictable by adding the monospecies profiles (Redanz et al., 2021). Host responses to polymicrobial plaque are therefore emergent properties of the consortium as well as of individual organisms.

Neutrophils form a major cellular barrier at the periodontal interface. Their phagocytosis, reactive oxygen species, granule enzymes and extracellular traps constrain microbial invasion, but excessive or manipulated responses can contribute to tissue injury. Neutrophils exposed to *S. oralis*, *P. gingivalis* or *Aggregatibacter actinomycetemcomitans* biofilms display different ROS, NET and MMP patterns (Mikolai et al., 2021). The relevant biological question is not whether neutrophils are protective or pathogenic in general, but whether recruitment and effector activity are appropriately regulated for the microbial challenge.

Periodontitis provides a striking example of microbial manipulation of host control. In a murine model, low-abundance *P. gingivalis* altered the quantity and composition of the resident microbiota and produced complement-dependent periodontal bone loss (Hajishengallis et al., 2011). Follow-up mechanistic work showed that C5aR-TLR2 crosstalk can blunt effective phagocytic clearance while leaving inflammatory signaling intact, effectively giving bystander protection to the surrounding community (Maekawa et al., 2014). These findings are consistent with keystone-like behavior in defined experimental models, but they do not imply that *P. gingivalis* alone accounts for human periodontitis.

Inflammation also changes the ecological conditions within the periodontal niche. Increased gingival crevicular fluid, vascular permeability, bleeding and tissue breakdown alter the supply of proteins, peptides and heme-containing compounds, conditions that can favor organisms adapted to proteolytic and asaccharolytic metabolism. Complement contributes to this amplification: microbial challenge can induce complement-dependent IL-6/IL-23 signaling and Th17 expansion (Wang et al., 2022). By contrast, DEL-1 limits LFA-1-dependent neutrophil recruitment and IL-17-associated bone loss, showing that effective host protection depends on regulated rather than maximal inflammation (Eskan et al., 2012).

## 6 Functional genomics and multi-omics for mechanistic discovery

Large oral-microbiome datasets provide extensive descriptive information, but measurement alone does not establish functional importance. A highly expressed gene may be dispensable, a metabolite associated with a pathogen may originate from another species, and a taxon can occupy a central position in a network without controlling community behavior. Mechanistic inference therefore requires perturbation in addition to observational profiling. **Table 2** summarizes the functional-genomic, multi-omic and spatial approaches discussed in this section and the next.

**Table 2:** Functional-genomic, multi-omics and spatial approaches applied to oral biofilm research.

Approach	Main question	Representative oral application	Principal strength	Major limitation	Ref.
Tn-seq	Which genes affect competitive fitness?	*S. mutans* essentiality/oral fitness; *F. nucleatum* coaggregation	Genome-scale functional selection	Public-good complementation; secondary mutations require validation	(Shields et al., 2018; Wu et al., 2021; Solano Morales et al., 2026)
CRISPRi	What happens when essential genes are partially depleted?	Inducible and arrayed *S. mutans* essential-gene resources	Access to non-deletable genes	General growth defects can mimic biofilm-specific phenotypes	(Shields et al., 2020; St. Pierre et al., 2024)
Metatranscriptomics	Which community genes are transcriptionally active?	Dentifrice intervention, caries surfaces, periodontal treatment	Community activity rather than DNA potential	Expression does not establish requirement	(Carda-Diéguez et al., 2022; Ev et al., 2023; Kobayashi et al., 2026)
Metaproteomics	Which microbial/host proteins are present?	Caries-associated plaque; plaque-regrowth interventions	Measures functional machinery closer to phenotype	Shared peptides and dynamic-range limitations	(Belda-Ferre et al., 2015; Rabe et al., 2022)
Integrated multi-omics	How do microbial and chemical layers covary?	Plaque proteomics/lipidomics/metabolomics/16S	Generates cross-layer hypotheses	Statistical integration requires experimental attribution	(Overmyer et al., 2021)
Spatial salivary metabolomics	How does chemistry vary by oral site/time?	Spatially and temporally resolved oral-fluid metabolome	Demonstrates sampling-site heterogeneity	Does not map metabolites inside intact plaque	(Ciurli et al., 2024)
Shotgun metagenomics	What genetic potential is present?	Multinational periodontitis; peri-implant disease	Species/strain and functional-potential resolution	DNA does not establish activity or causality	(Alexandre et al., 2025; Ghensi et al., 2025)
Multiplex spatial imaging	Who is adjacent to whom?	Micron-scale plaque biogeography and caries architecture	Preserves spatial organization	Limited molecular breadth relative to sequencing	(Mark Welch et al., 2016; Kim et al., 2020)
pH/O2 imaging	What microenvironment do cells experience?	Human/*in vitro* pH and oxygen gradients	Direct physiological measurement	Limited molecular attribution	(Von Ohle et al., 2010; Kristensen et al., 2023; Lynge et al., 2026)
Flow/microfluidic models	How do physical conditions influence biofilms?	Plaque-on-chip and sensor-integrated systems	Controlled flow and spatial imaging	Still simplified relative to the mouth	(Lemus and Valm, 2023; Makkar et al., 2023; Debener et al., 2024)
Genome-scale metabolic models	Which metabolic reactions/exchanges are feasible?	*P. gingivalis*, *S. mutans*, *S. oralis*, oral communities	Mechanistically constrained hypotheses	Depends on model completeness and assumed medium	(Mazumdar et al., 2009; Bernstein et al., 2019; Jijakli and Jensen, 2019; Jensen et al., 2020; Pucci et al., 2026)
Machine learning	Can microbial features predict disease/outcome?	Caries and periodontitis classifiers	Captures multivariate patterns	External transportability and causality remain limitations	(Grier et al., 2021; Wei et al., 2025; Seo et al., 2026; Xie et al., 2026)

Biofilm resilience is best treated as a trajectory rather than a single end point. Multi-omics can help separate the immediate response to perturbation from the subsequent recovery phase by following transcriptional activity, protein abundance, metabolite output and community composition during treatment and after withdrawal. Discordance among these layers is informative: taxonomic stability can coexist with functional change, while apparent compositional recovery may conceal persistent transcriptional or metabolic reprogramming. Recent systems-level perspectives therefore argue for longitudinal, integrative measurements when resilience is the biological question (Ranganathan et al., 2026).

Transposon sequencing is one of the most direct routes from genotype to fitness. A dense *S. mutans* UA159 Tn-seq library turned up essential genes plus additional loci needed for sustained growth, and an *in vivo* oral challenge exposed fitness determinants relevant to host persistence (Shields et al., 2018). What Tn-seq gives you is a prioritized gene list, not an explanation of why disrupting those genes changes fitness, a gap made concrete by the *F. nucleatum* CarRS-RadD work, where the transposon screen produced candidates, but it took separate biochemical and binding experiments to pin down the lysine-regulated adhesion mechanism (Wu et al., 2021).

CRISPR interference pushes functional analysis into territory Tn-seq cannot reach: genes that cannot simply be deleted. The endogenous *S. mutans* Cas9 system has been repurposed for tunable transcriptional repression (Shields et al., 2020), and an arrayed SNAP CRISPRi resource now targets more than 250 essential or growth-supporting genes (St. Pierre et al., 2024). Because strong repression of an essential gene can cause generalized growth arrest, biofilm specificity must be distinguished from nonspecific fitness loss through titration, planktonic controls and phenotype-specific measurements.

Arrayed mutant resources address another limitation of pooled screening: extracellular products from neighboring mutants can mask defects in matrix synthesis, secreted enzymes, metabolites or signals. A 2026 sequence-defined *S. mutans* collection of 9,216 transposon mutants enabled individual screening and revealed previously unrecognized candidate biofilm determinants (Solano Morales et al., 2026). Whole-genome sequencing also showed that 25% of selected biofilm-defective mutants carried spontaneous recombination at the *gtfBC* locus, while another subset had lost TnSmu1 (Solano Morales et al., 2026). Whole-genome verification is therefore necessary before assigning an extreme biofilm phenotype solely to the intended transposon insertion.

Metatranscriptomics adds community activity to the picture. Paired metagenomic/metatranscriptomic profiling during dentifrice intervention separated changes in community composition from changes in transcriptional activity (Carda-Diéguez et al., 2022). In a small study of five caries-active individuals, metatranscriptomics across sound, active and arrested surfaces suggested that some functional features were shared across sites within individuals, cautioning against overgeneralization from a single lesion (Ev et al., 2023). Longitudinal periodontal metatranscriptomics further showed that clinical improvement after therapy can accompany transcriptional and network changes without complete convergence toward a healthy reference state (Kobayashi et al., 2026). Expression nevertheless remains an activity marker, not proof of functional necessity.

Metaproteomics measures the machinery actually present. Human dental plaque studies identify microbial and host proteins associated with caries and metabolic functions (Belda-Ferre et al., 2015), while plaque-regrowth interventions demonstrate that treatments can alter the active protein landscape differently even when they all affect plaque accumulation (Rabe et al., 2022). Peptide sequences shared across taxa, low-abundance proteins that fall below detection, and the sheer dominance of host protein in the sample all remain significant limitations of the approach.

Multi-omics is most informative when one data layer is used to test or constrain interpretations from another. In dental plaque, cross-omic analysis associated *Lautropia* with monomethyl phosphatidylethanolamine; the investigators then cultured *Lautropia mirabilis* and directly confirmed lipid production (Overmyer et al., 2021). This progression from association to a specific hypothesis and experimental attribution provides a useful model for multi-omics studies.

Spatially resolved salivary sampling has shown that the oral metabolome varies substantially by anatomical site and over time (Ciurli et al., 2024). This finding is relevant to sampling design, but it is not a map of metabolite distributions within intact dental plaque. Shotgun metagenomics addresses a different question by cataloguing genetic potential. In a multinational periodontitis study, subgingival taxonomic composition varied markedly by geography even though disease-associated functional patterns were comparatively more conserved (Alexandre et al., 2025), while peri-implant metagenomics has improved species- and function-level resolution of disease-associated plaque (Ghensi et al., 2025). Even with this added resolution, DNA describes potential rather than the activity of the community at the time of sampling.

Spatial imaging preserves information that is lost when plaque is homogenized for bulk analysis. Multiplex fluorescence methods directly resolve micron-scale plaque biogeography (Mark Welch et al., 2016), and imaging of cariogenic biofilms links *S. mutans*-rich structures with acidic microenvironments (Kim et al., 2020). Flow-based models then bridge the gap between sequencing and physiology: human-plaque culture systems run under salivary-like flow can be paired with multispectral imaging (Lemus and Valm, 2023), and sensor-integrated multispecies chambers allow local pH monitoring alongside confocal/FISH measurements (Debener et al., 2024).

Several distinctions are worth holding onto throughout this literature. Presence is not the same as activity, nor is activity the same as necessity; association does not amount to exchange, spatial proximity is not molecular recognition, fitness is not mechanism, essentiality does not guarantee therapeutic selectivity, and a prediction is not a validation. The most convincing oral-biofilm studies do not settle for one method: they move back and forth between community profiling, functional genetics, biochemical experiments, spatial methods and increasingly realistic multispecies or host-associated models.

## 7 Computational integration and predictive oral biofilm biology

Computational analysis has become close to essential simply because oral biofilm datasets now span hundreds of taxa, thousands of genes and transcripts, large metabolite inventories, spatial measurements and longitudinal clinical variables at once. How useful it is depends on keeping each method’s output clearly separate from the biological concept it only approximates.

Association networks can reveal phenotype-related structure that is not apparent from differential abundance alone. In caries, compositionally aware metatranscriptomic networks showed substantial restructuring between phenotypes despite relatively modest differential abundance (Espinoza et al., 2022). What these networks capture, though, is statistical association, not direct microbial interaction.

Genome-scale metabolic models provide a more mechanistically constrained framework. A *P. gingivalis* model reproduced several known aspects of amino-acid metabolism and generated candidate vulnerabilities (Mazumdar et al., 2009). The iSMU model of *S. mutans* linked 675 reactions to 493 genes and was iteratively tested against carbon-source use, nutrient requirements and gene essentiality; model-generated hypotheses about sorbitol and complex-carbohydrate metabolism were then validated experimentally (Jijakli and Jensen, 2019). A corresponding *S. oralis* reconstruction achieved substantial but incomplete agreement with phenotype and essentiality data, revealing both the utility and the limits of model completeness (Jensen et al., 2020).

Community-scale modeling extends this approach. Metabolic network analysis across hundreds of oral organisms predicted differences in biosynthetic capacity and potential dependencies (Bernstein et al., 2019). A 2026 infant oral-microbiome study combined metagenome-assembled genomes with genome-scale models to generate candidate exchanges between previously undescribed co-occurring *Streptococcus* and *Rothia* species (Pucci et al., 2026). Such exchanges are predictions until validated by metabolite measurements, isotope tracing, supplementation or pathway disruption.

Machine learning can identify multivariate signatures of disease. In a longitudinal early-childhood-caries cohort, microbiome composition predicted subsequent disease with moderate discrimination before clinical onset and stronger discrimination closer to diagnosis (Grier et al., 2021). Larger multi-dataset studies show that performance varies by age group, specimen type and algorithm (Wei et al., 2025). External validation is therefore critical. A large 2026 oral-rinse study developed a periodontitis classifier with independent validation and used SHAP values to identify model-driving taxa (Xie et al., 2026), whereas another 2026 periodontitis study achieved very high internal performance but substantially variable results across three external datasets (Seo et al., 2026). Internal AUC alone is therefore a poor measure of clinical transportability.

Microbiome machine-learning studies also carry limitations that arise before model choice. Compositional data can distort ordinary correlation structure; sequencing platform, extraction and preprocessing differences can introduce batch effects; and sample numbers are often small relative to the feature space. Repeated samples from the same donor create an additional risk of leakage if training and test sets are split at the sample rather than participant level. Geographic and clinical domain shift can then make apparently stable internal features fail in external cohorts. Feature attribution should therefore be evaluated for stability across resampling and cohorts, and external validation should be designed at the donor or cohort level whenever the sampling structure allows it.

Explainable-AI terminology should be used sparingly. SHAP values and related feature-importance methods explain why a fitted model made a prediction relative to its training distribution; they do not demonstrate that increasing or decreasing the featured organism will change disease. Current oral evidence more strongly supports interpretable microbiome classification than mechanistically explainable AI. A biologically meaningful workflow is predictive model -> stable feature across cohorts -> pathway hypothesis -> targeted perturbation -> experimental confirmation or rejection.

Structure-based screening shows how computational prioritization can be linked to experiment. Screening against *S. mutans* GtfC identified compounds that were subsequently evaluated by binding, enzyme, biofilm and animal assays (Zhang et al., 2017). A 2026 drug-repurposing study similarly moved from structural screening to biochemical binding, enzymatic assays, confocal biofilm analysis and genetic testing with gtf deletion strains; the biomass-reducing effect of radotinib was strongly dependent on GtfC in the tested systems (De Lucena et al., 2026). These studies illustrate a hierarchy of evidence: docking < predicted pose or simulation stability < direct binding < enzyme inhibition < cellular phenotype < genetic target validation < multispecies validation < *in vivo* validation. **Figure 3** situates these same steps within the broader systems workflow, connecting initial observation and computational prioritization to experimental validation.

**Figure 3:**
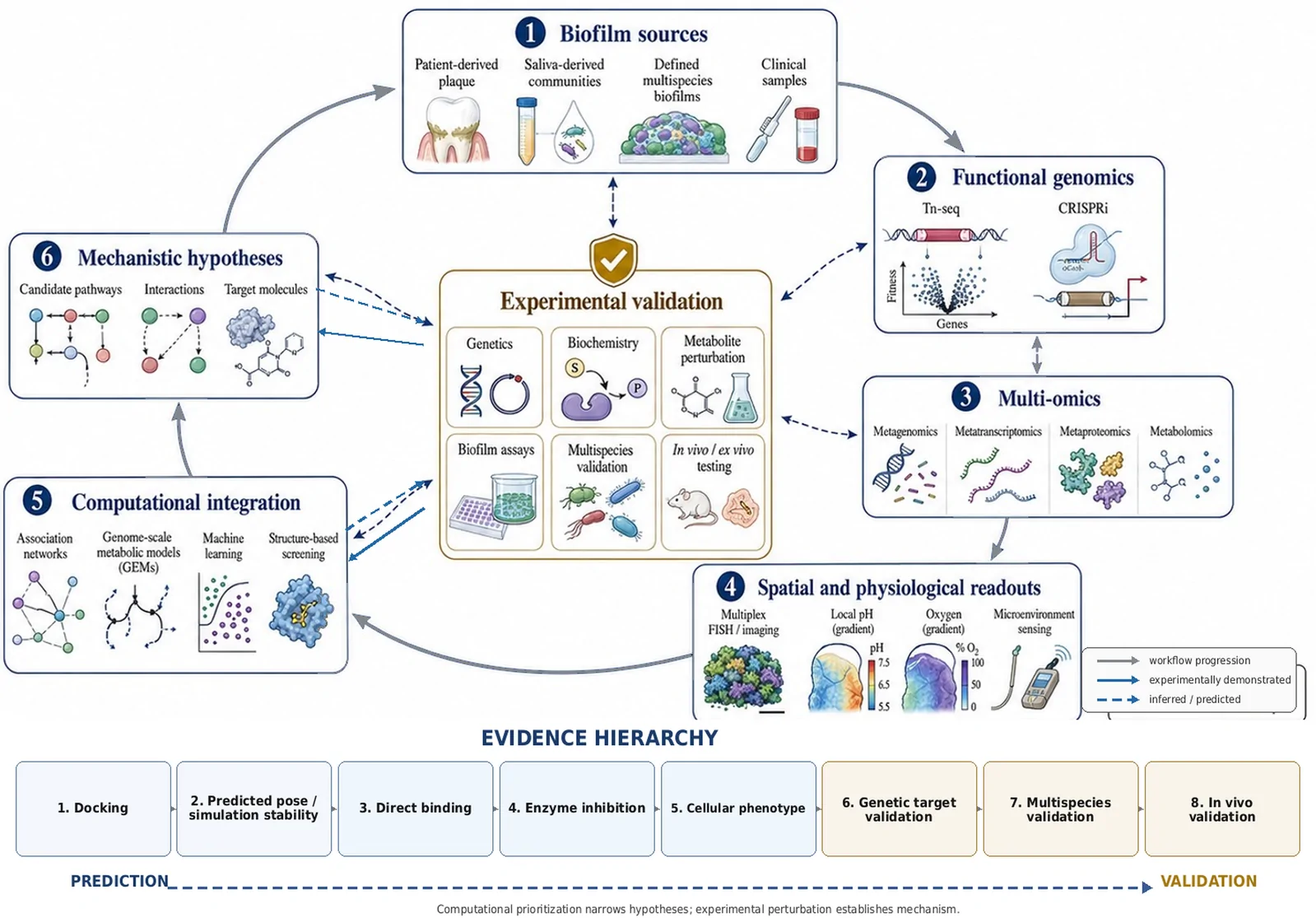
Systems workflow from oral biofilm observation to experimentally validated vulnerability. This image is an original graphical synthesis prepared for this review; the relationships it depicts are drawn from the cited primary literature. Evidence status is encoded visually: solid arrows for experimentally demonstrated relationships, dashed arrows for inferred or predicted links, and a distinct validation bar separating prediction from biochemical, genetic, multispecies and *in vivo* confirmation. Patient-derived or defined biofilms lend themselves to characterization by Tn-seq/CRISPRi (Shields et al., 2018; Wu et al., 2021; Solano Morales et al., 2026), metagenomics (Alexandre et al., 2025; Ghensi et al., 2025), metatranscriptomics (Carda-Diéguez et al., 2022; Ev et al., 2023; Kobayashi et al., 2026), metaproteomics (Belda-Ferre et al., 2015; Rabe et al., 2022), metabolomics (Ciurli et al., 2024), spatial imaging (Mark Welch et al., 2016) and physiological measurements such as local pH and oxygen (Von Ohle et al., 2010; Kristensen et al., 2023; Lynge et al., 2026). Computational integration turns this input into candidate mechanisms or targets (Mazumdar et al., 2009; Bernstein et al., 2019; Jijakli and Jensen, 2019; Jensen et al., 2020; Grier et al., 2021; Overmyer et al., 2021; Espinoza et al., 2022; Wei et al., 2025; Pucci et al., 2026; Seo et al., 2026; Xie et al., 2026), which then require targeted genetic, biochemical, metabolic or pharmacological perturbation and validation in multispecies and host-associated models (Zhang et al., 2017; Lemus and Valm, 2023; Makkar et al., 2023; Debener et al., 2024; De Lucena et al., 2026). The central validation sequence explicitly separates prediction from experimental confirmation and follows the hierarchy docking < predicted pose or simulation stability < direct binding < enzyme inhibition < cellular phenotype < genetic target validation < multispecies validation < *in vivo* validation.

Several distinctions are essential when interpreting computational studies of oral biofilms. Correlation does not establish microbial interaction, network centrality does not identify a keystone organism by itself, and a model-predicted metabolite exchange is not evidence that cross-feeding occurs *in vivo*. Likewise, SHAP values describe the contribution of features to model predictions rather than virulence mechanisms. Docking scores do not measure binding affinity, and stability in a molecular-dynamics simulation does not establish target engagement. Computational methods are most useful when they narrow the experimental search space and generate hypotheses that can be tested directly.

## 8 Emerging precision anti-biofilm strategies

Broad-spectrum antimicrobials have clear limitations in a community where disease-associated functions coexist with health-associated organisms. This has shifted attention toward interventions that act on a defined process, organism or microenvironment. The term “precision,” however, should be used only when selectivity or ecological localization has been demonstrated. **Table 3** summarizes the representative interventions discussed below and the highest level of evidence available for each.

**Table 3:** Emerging anti-biofilm interventions and current evidence level.

Strategy	Target/process	Representative evidence	Highest evidence tier/supported outcome	Key limitation	Ref.
Adhesion-blocking peptide	Antigen I/II-mediated attachment	Peptide dentifrice reduced plaque mutans streptococci	Tier 4 - human proof-of-mechanism; inhibition of attachment/target abundance	No demonstrated caries-incidence benefit	(Li et al., 2009)
STAMP C16G2	Selective *S. mutans* killing	C16G2 human proof-of-mechanism; newer targeted peptides extend selective *in vitro* antibiofilm activity	Tier 4 - human proof-of-mechanism; selective suppression and disease-proximal protection	Long-term ecological/clinical durability unresolved	(Sullivan et al., 2011; Guo et al., 2015; Wei et al., 2026)
Species-biased small molecules	*S. mutans* growth/biofilm fitness	Selectivity against tested commensal streptococci	Tier 3 - animal/preclinical; inhibition and disease modification in model systems	Limited microbiome breadth tested	(Liu et al., 2011; Garcia et al., 2017)
Gtf inhibition	Glucan/EPS synthesis	Small-molecule and structure-guided Gtf inhibition	Tier 3 - animal/preclinical; matrix-synthesis inhibition and caries reduction	Ecological consequences and human efficacy unresolved	(Ren et al., 2016; De Lucena et al., 2026)
Matrix-degrading enzymes	Glucans/eDNA	Mutanase/dextranase and dextranase studies	Tier 3 - animal for selected enzymes; disruption of matrix/biofilm structure	Stability, penetration and viable-cell fate	(Jiao et al., 2014; Liao et al., 2014; Otsuka et al., 2015)
Quorum interference	Peptide/AI-associated signaling	Quorum-quenching extracts; ComA inhibitor	Tier 1 - *in vitro*; signaling inhibition and reduced biofilm formation	Non-bactericidal does not equal ecologically selective	(Ishii et al., 2017; Muras et al., 2018)
Catalytic nanoparticles	Acidic biofilm microenvironment/matrix	Ferumoxytol and dextran-coated iron oxide nanozymes	Tier 3 - animal; environment-responsive disruption and caries reduction	Human long-term safety and ecology unknown	(Liu et al., 2018; Naha et al., 2019)
Probiotic modulation	Competition/metabolic/host effects	*S. dentisani* trial; *L. reuteri* periodontal RCT	Tier 5 - controlled human trials; ecological/clinical modulation with indication-dependent efficacy	Efficacy is strain- and indication-dependent	(Ferrer et al., 2020; Bujaldón et al., 2026)
Antimicrobial photodynamic therapy	Local reactive-species generation	Positive short-term RCTs; uncertain pooled long-term benefit	Tier 5 - controlled human trials/systematic synthesis; adjunctive disease modification with uncertain durable benefit	Protocol heterogeneity; low-certainty durable benefit	(Jervøe-Storm et al., 2024; Nie et al., 2024)
Antibiofilm restorative materials	Surface-associated accumulation	Human *in-situ* QADM; newer animal-tested composites	Tier 4 - human *in situ* plus animal; inhibition of surface-associated biofilm accumulation	Activity may decline with mature conditioning layers	(Melo et al., 2018; Lin et al., 2026)

Therapeutic evidence tiers: Tier 1, biochemical/monospecies *in vitro*; Tier 2, defined multispecies or ex vivo; Tier 3, animal efficacy; Tier 4, human proof-of-mechanism/*in situ* evidence; Tier 5, controlled clinical outcome or systematic clinical evidence. A higher tier indicates model proximity to clinical use, not necessarily higher certainty or larger benefit.

The precision framework introduced in Section 1 is used here to separate species selectivity, mechanistic targeting, microenvironmental localization and ecological sparing. None should be inferred from a known molecular target alone. Because candidate interventions occupy very different evidence levels, the discussion below is paired with the translational bottlenecks in Section 9 rather than treating preclinical biofilm effects as proxies for clinical benefit.

One option is to interfere with initial attachment. In a human study, a dentifrice containing a peptide derived from streptococcal antigen I/II reduced plaque mutans streptococci (Li et al., 2009). This is clinical proof-of-mechanism for receptor-level interference, but not evidence of reduced caries incidence. Specifically targeted antimicrobial peptides (STAMPs) provide a more direct strategy. In a pilot human intra-oral model, the STAMP C16G2 reduced plaque and salivary *S. mutans*, lowered lactic-acid production and limited enamel demineralization while total bacterial levels changed relatively little (Sullivan et al., 2011). In saliva-derived communities, selective depletion of *S. mutans* also altered the abundance of other members during recovery (Guo et al., 2015), demonstrating that targeted killing can reorganize the community rather than leave it unchanged.

Broader antimicrobial-peptide and antibody literature supports the feasibility of highly selective recognition and membrane- or antigen-directed killing, although evidence from ESKAPE pathogens should be treated as contextual rather than oral-specific support (Nagarajan et al., 2024). Recent oral work continues to extend this concept: a 2026 chimeric peptide designed to target *S. mutans* showed strong *in vitro* selectivity over tested commensal streptococci and disrupted established biofilms, but the study remains preclinical and does not establish ecological stability or clinical prevention (Wei et al., 2026).

Species-biased small molecules provide related preclinical evidence. Certain 2-aminoimidazole/benzimidazole compounds inhibit *S. mutans* more strongly than tested commensal streptococci (Liu et al., 2011), and the small molecule 3F1 disrupts *S. mutans* biofilms in preclinical models (Garcia et al., 2017). Selectivity against a few laboratory strains should not, however, be equated with complete microbiome sparing.

Matrix synthesis can be targeted without primarily killing bacteria. Small-molecule inhibition of *S. mutans* glucosyltransferases reduces EPS production, biofilm virulence and caries in rodent models (Ren et al., 2016), while the structure-guided radotinib study provides recent evidence for GtfC-dependent antibiofilm activity (De Lucena et al., 2026). Enzymatic degradation provides another route. Chimeric mutanase-dextranase systems reduce glucan formation and degrade glucan-rich biofilms *in vitro* (Otsuka et al., 2015), while a marine-derived dextranase reduced *S. mutans* biofilm features and caries severity in an animal model (Jiao et al., 2014). DNase similarly targets eDNA-associated structure (Liao et al., 2014). Matrix disruption may be particularly valuable in combination therapy because it can expose embedded cells to mechanical clearance or a second antimicrobial treatment.

Quorum-associated pathways are attractive because signaling can be perturbed without direct killing. Quorum-quenching extracts from *Tenacibaculum* sp. reduced *S. mutans* biofilm formation without substantially inhibiting growth but also affected other streptococci, so the effect was not species-specific (Muras et al., 2018). A chemically defined ComA peptidase inhibitor reduced *S. mutans* competence-associated signaling and biofilm formation without detectable growth inhibition (Ishii et al., 2017). Non-bactericidal, however, does not mean ecologically neutral; signaling pathways can influence competition, gene transfer and interspecies relationships.

Quorum interference and antivirulence should not be treated as interchangeable categories. Competence signaling, AI-2-associated phenotypes, adhesin blockade, matrix inhibition and direct virulence-factor suppression act at different biological levels and may impose different selection pressures. It is often proposed that non-bactericidal antivirulence strategies could reduce resistance selection relative to lethal exposure, but this remains poorly tested for most oral-biofilm interventions; altered receptors, compensatory pathways or community replacement could still erode efficacy during repeated exposure.

Catalytic nanoparticles exploit a different form of precision: the disease-associated microenvironment. Ferumoxytol exhibits peroxidase-like activity in acidic cariogenic biofilms and, with hydrogen peroxide, damages bacterial membranes and matrix components; efficacy has been shown ex vivo and in rodent caries models (Liu et al., 2018). Dextran-coated iron oxide nanozymes improve localization and pH-responsive activity and reduce caries severity in animals without obvious gingival toxicity under the tested conditions (Naha et al., 2019). These data support environment-responsive preclinical activity, not established human microbiome safety.

Probiotics aim to alter ecology rather than remove a target. In a randomized human study, topical *Streptococcus dentisani* increased salivary ammonia and calcium and altered plaque-associated parameters and community composition (Ferrer et al., 2020), but caries incidence was not tested. Conversely, a 2026 randomized trial of *Limosilactobacillus reuteri* as an adjunct to periodontal instrumentation in patients with diabetes found no significant additional periodontal clinical or microbiological benefit (Bujaldón et al., 2026). Probiotic efficacy is therefore strain-, indication- and protocol-dependent.

Antimicrobial photodynamic therapy (aPDT) has reached human testing. A 2024 split-mouth randomized trial reported short-term probing-depth and subgingival-microbiome changes after repeated adjunctive aPDT (Nie et al., 2024). The broader evidence is less secure: a 2024 Cochrane review of 50 randomized trials found very-low-certainty evidence for clinically important six-month benefit (Jervøe-Storm et al., 2024). aPDT should therefore be described as biologically active with mixed clinical evidence rather than as an established superior adjunct.

Bioactive restorative materials provide continuous local intervention. A human *in-situ* study of QADM-containing composite showed stronger effects during early biofilm formation than after 14 days of maturation (Melo et al., 2018). A 2026 quaternary-ammonium-modified mesoporous-silica composite reduced *S. mutans*-associated biofilm and secondary caries in an animal model while retaining several measured material properties (Lin et al., 2026). Human durability and recurrent-caries prevention remain untested.

Combination therapy may be more realistic than a single “magic bullet.” A matrix-degrading agent can increase access to embedded cells; a targeted antimicrobial can then remove a defined organism; an alkalinizing or ecological intervention can reduce the environmental pressure that favors recolonization. Combination therapy should mean mechanistic complementarity rather than simply adding several antimicrobials. Successful treatment should also be defined by the relevant endpoint (matrix architecture, local pH, virulence, ecological stability, tissue protection or clinical disease), not total biomass alone. **Figure 4** depicts this transition from indiscriminate microbial killing toward mechanism- and ecology-informed biofilm control.

**Figure 4:**
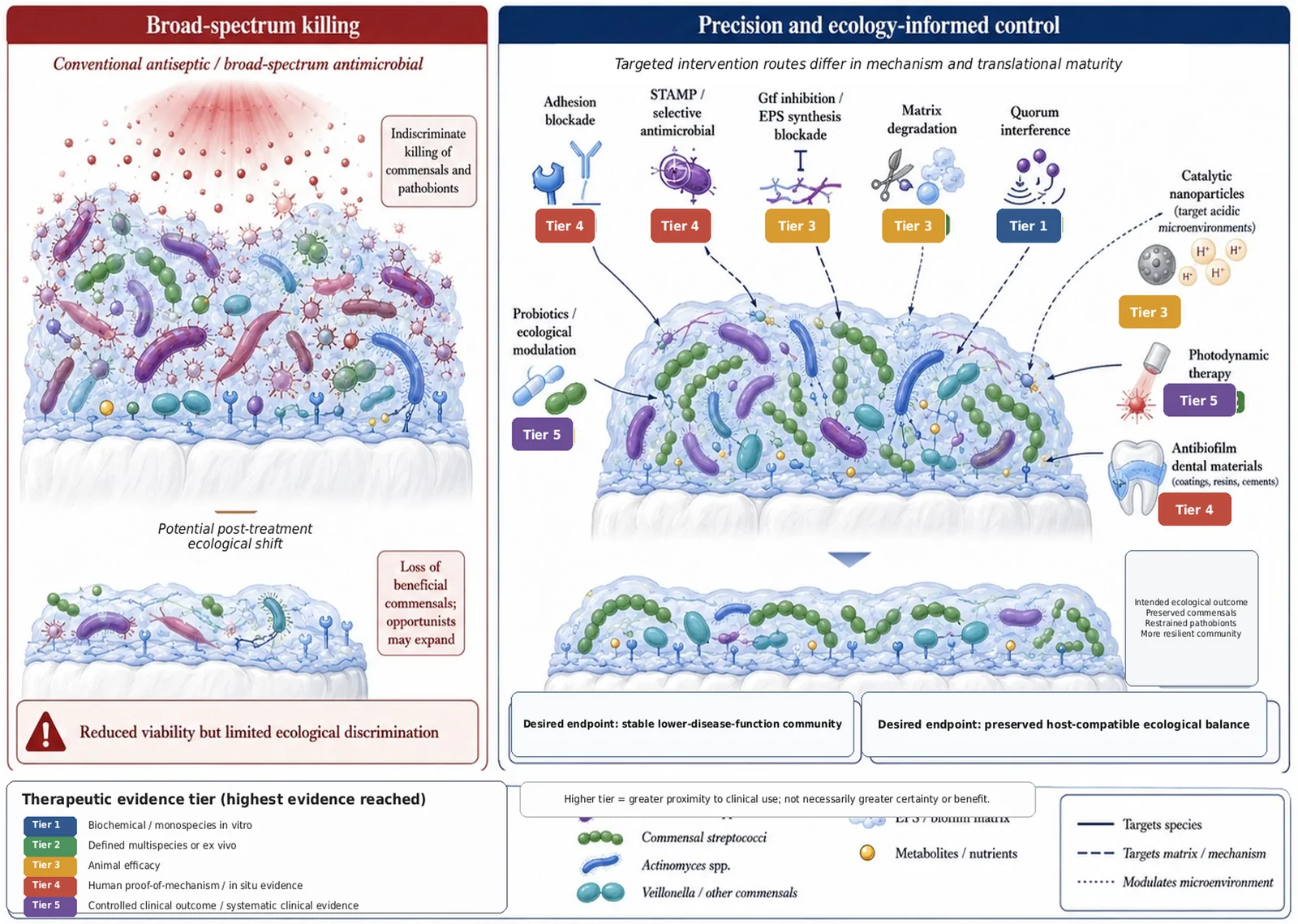
Therapeutic transition from indiscriminate microbial killing to mechanism- and ecology-informed biofilm control. This image is an original graphical synthesis prepared for this review; the relationships it depicts are drawn from the cited primary literature. Evidence labels distinguish *in vitro*, ex vivo, animal, human proof-of-mechanism and clinical-outcome evidence so that schematic proximity does not imply equivalent translational maturity. Conventional broad-spectrum treatment mainly reduces microbial viability, with little discrimination between disease-associated and health-associated organisms. Precision approaches instead intervene at defined vulnerabilities: adhesion (Li et al., 2009; Sullivan et al., 2011), species identity (Liu et al., 2011; Garcia et al., 2017), EPS synthesis (Ren et al., 2016; De Lucena et al., 2026), matrix integrity (Jiao et al., 2014; Liao et al., 2014; Otsuka et al., 2015), signaling (Ishii et al., 2017; Muras et al., 2018), acidic chemistry (Liu et al., 2018; Naha et al., 2019), microbial competition (Ferrer et al., 2020; Bujaldón et al., 2026) or the colonized material surface (Melo et al., 2018; Lin et al., 2026). Evidence levels range from *in vitro* studies to human trials and are displayed separately so that schematic proximity does not imply equivalent translational maturity. The image contrasts broad-spectrum killing with multiple targeted routes toward a stable community with lower disease-associated function.

## 9 Major controversies, translational bottlenecks and unresolved questions

Four related outcomes should be kept separate when antibiofilm effects are described. Suppression denotes reduced abundance or activity of a target without requiring major structural change; inhibition refers to prevention or reduction of establishment or activity during exposure; disruption describes loss of established architecture or matrix integrity even when viable cells remain; and eradication should be reserved for elimination of viable organisms to a defined detection threshold. Ecological reprogramming is a persistent shift to a different community state, whereas ecological restoration requires recovery toward a health-compatible functional state supported by ecological and/or host-relevant measurements. Suppression or disruption, by themselves, do not establish restoration.

The growing mechanistic detail available for oral biofilms has not removed several basic uncertainties about how disease-associated communities should be interpreted or controlled. In many cases, apparent disagreement reflects measurement at different biological levels. Reduction of one pathobiont is not the same as restoration of community function; reduced biomass is not equivalent to reduced tissue damage; and computational centrality is not necessarily causal control. **Table 4** summarizes these major unresolved questions and corresponding experimental priorities.

**Table 4:** Major unresolved questions and experimental priorities.

Knowledge gap	Why it matters	Current limitation	Priority experiment/approach	Translational implication
Is a proposed keystone organism causal?	Association does not demonstrate community control	Cross-sectional studies dominate	Selective depletion and restoration in increasingly complex models	Avoids targeting biomarkers rather than drivers
Does selective killing restore health?	Removing a species can reorganize the community	Short follow-up	Post-treatment recovery and functional profiling	Tests ecological durability
Are signaling inhibitors ecologically selective?	Non-lethal perturbation can alter competition	Monospecies reporter assays	Multispecies longitudinal signaling studies	Defines collateral ecological effects
Is matrix disruption sufficient?	Viable dispersed cells may recolonize	Matrix often measured without cell fate	Degradation plus clearance/recolonization experiments	Supports rational combinations
Which model best predicts efficacy?	Model choice can change outcome	Static simplified screens	Staged monospecies to defined community to microcosm to host model	Improves translational relevance
How important is donor variability?	Starting ecology may determine treatment response	Pooling masks response heterogeneity	Multi-donor microcosms with donor-stratified analysis	Supports responder selection
Are computational hubs therapeutic targets?	Centrality is not causality	Limited perturbation	Prediction followed by genetics/metabolic/spatial validation	Converts prediction into mechanism
What defines successful treatment?	Biomass, viability and disease are non-equivalent	Incompatible surrogate endpoints	Molecular-to-clinical endpoint hierarchy	Improves study comparability
Are effects durable after withdrawal?	Biofilms rapidly recolonize	Immediate readouts predominate	Recovery and repeated-challenge phases	Distinguishes suppression from reprogramming
How should ecological safety be defined?	Diversity alone does not represent function	Sparse functional safety data	Composition plus metabolism plus host-response analysis	Evidence for genuine precision therapy

As introduced in Section 5, low-abundance *P. gingivalis* can alter resident microbiota and drive complement-dependent periodontal bone loss in murine models (Hajishengallis et al., 2011; Maekawa et al., 2014). This keystone-pathogen behavior does not imply that *P. gingivalis* alone explains every case of human periodontitis. The more defensible model is that some organisms or functions can exert disproportionate influence, but the magnitude and direction of that influence depend on surrounding community and host context (Lamont and Hajishengallis, 2015; Hajishengallis and Lamont, 2021). Causality therefore requires perturbation: selective suppression or removal should produce a predictable community and disease phenotype, and restoration should recover at least part of that phenotype.

Targeted killing does not automatically restore a “healthy microbiome.” After *S. mutans* depletion, C16G2 also changes the structure of the surrounding community (Guo et al., 2015), and periodontal communities may remain transcriptionally distinct from health even after clinical improvement (Kobayashi et al., 2026). Given how much healthy oral microbiomes differ from one individual to the next (Hajishengallis et al., 2023; Dieckow et al., 2024; Alexandre et al., 2025), chasing a single universal taxonomic endpoint is probably unrealistic. Functional criteria look more promising: reduced sustained acidification, restored alkali-generating capacity, and suppression of proteolytic inflammatory ecology.

Quorum interference raises a comparable issue. LuxS/AI-2-dependent interactions do occur in some oral systems (McNab et al., 2003; Rickard et al., 2006), but AI-2 is not a conserved quorum-sensing mechanism across *F. nucleatum* (G C et al., 2026), and peptide systems such as ComCDE/ComRS regulate competence, stress and bacteriocin-associated functions (Li et al., 2002; Shields and Burne, 2016; Kaspar et al., 2017) rather than a single unified pathway. A signaling inhibitor can therefore be non-bactericidal and still reshape competition or horizontal gene transfer, which means its community-level consequences need to be measured directly, not assumed from its mechanism of action.

Matrix disruption on its own is also unlikely to be enough. Oral matrices are built from glucans, eDNA, proteins and vesicle-associated material, and how much each contributes shifts as the biofilm ages (Liao et al., 2014; Otsuka et al., 2015; Yarmola et al., 2022). Degrading one component can expose cells to clearance, true, but it can equally release viable bacteria that simply recolonize afterward. Matrix destabilization is better treated as one step in a longer sequence than as a therapeutic endpoint in itself.

Transferability across models remains a major bottleneck in this field. Monospecies systems are indispensable for pinning down mechanism, but interspecies interactions can shift the phenotype substantially: GtfB binds *C. albicans* (Hwang et al., 2017), *V. parvula*-*S. gordonii* adhesins reshape mixed-biofilm architecture (Dorison et al., 2024), and *S. oralis*-derived H2O2 can alter *S. mutans*-associated disease phenotypes (Kim et al., 2022). A staged validation pathway is far more defensible than jumping straight from one extreme to the other: monospecies mechanism -> defined multispecies interaction -> patient-derived microcosm -> host-associated/ex-vivo system -> animal or human testing.

Model realism should be hypothesis driven. Static microplates omit flow and many gradients, but adding complexity is valuable only when the added feature changes the biology being tested. A gingival crevice-on-chip showed that simulated crevicular-fluid flow altered bacterial clearance and inflammatory output (Makkar et al., 2023). Likewise, pH and oxygen gradients are prominent features of mature biofilms (Von Ohle et al., 2010; Kristensen et al., 2023; Lynge et al., 2026). Flow, shear, oxygen, host tissue or enamel substrate should therefore be incorporated when the mechanism depends on them, rather than as decorative engineering complexity.

Donor variability is biological information, not merely noise. Subgingival microcosm biofilms cluster strongly by inoculum source, donor and periodontal status (Fernandez Y Mostajo et al., 2017). A 2025 ex-vivo screening model similarly showed reproducible treatment patterns alongside genuine donor-dependent response variation (Adami et al., 2025). Human implant biofilms are also highly subject-specific (Dieckow et al., 2024), and machine-learning classifiers can lose performance across external cohorts (Seo et al., 2026). Multi-donor experiments and donor-stratified analyses should become standard when the conclusion is intended to generalize beyond one starting microbiome.

Outcome selection is another unresolved problem. Biofilm biomass, viable counts, matrix volume, pH, species abundance, tissue demineralization and clinical endpoints describe different properties. Engineered salivary peptides reduced enamel demineralization without significantly reducing *S. mutans* biofilm biomass (Marin et al., 2022). If biomass alone had been used, a disease-relevant protective effect would have been missed. Conversely, a large short-term reduction in viable counts may be followed by rapid recovery if environmental selection remains unchanged.

A useful endpoint hierarchy is therefore: molecular target engagement -> microbial viability or expression -> biofilm architecture and metabolism -> ecological composition and resilience -> host/tissue outcome -> clinical disease. Evidence at one level should not automatically be described as success at the next.

Persistence and recolonization are particularly undermeasured. Oral treatment is intermittent, surfaces are rapidly recoated by saliva, nutrient pulses recur and untreated oral reservoirs continuously seed new cells. Intervention studies should include a recovery phase after withdrawal and ask whether the community returns to its original state, enters a new stable state or simply rebounds once the drug is removed.

Precision therapy brings its own safety questions along with it. Removing a pathobiont can disrupt metabolic interactions that are still poorly characterized, quorum interference can alter competence or gene transfer, and matrix enzymes can release cells or other biologically active material into the surrounding environment. Nanoparticles may be repeatedly swallowed or retained in tissues. Surface-bound antimicrobials can lose effectiveness as conditioning films and mature matrices accumulate (Melo et al., 2018). Ecological safety should therefore include composition, function and host response rather than alpha diversity alone.

### 9.1 Evolutionary adaptation under antibiofilm selection

Selective pressure can change an intervention’s performance even when the initial target is well defined. Resistance is a heritable reduction in susceptibility, whereas tolerance and persistence describe survival phenotypes that need not involve a stable change in MIC; these states can nevertheless prolong exposure and create opportunities for resistant variants to emerge (Liu et al., 2024). In a multispecies biofilm, adaptation can also occur ecologically: a targeted organism may be replaced by another taxon, a community partner may supply a redundant function, or changes in matrix production and receptor expression may reduce access to a single-target therapy. For many precision oral interventions, the rate and direction of such adaptation remain unknown. Serial-passage experiments, repeated treatment-withdrawal cycles and long-term multispecies selection studies should therefore be incorporated earlier in preclinical development rather than assuming that target selectivity guarantees evolutionary durability.

Standardization is needed, but should not erase biologically important complexity. Oral biofilm studies should report microbial source and donor/strain identity, substrate and surface conditioning, medium, atmosphere, flow/shear, inoculation, biofilm age, nutrient challenge, treatment exposure, recovery conditions and at least one structural or functional endpoint in addition to biomass. Beyond this core, variables should be selected according to the disease process being modeled.

The central translational gap is between biofilm control and disease control. Many interventions modify an experimental biofilm; far fewer show durable prevention or treatment of human disease. The most defensible pathway is defined molecular mechanism -> reproducible biofilm phenotype -> multispecies validation -> patient-derived community validation -> host/tissue compatibility -> *in vivo* efficacy -> human proof-of-mechanism -> adequately powered clinical outcome trial. The unresolved challenge is not simply to discover stronger antibiofilm agents, but to determine which biofilm property must change, in which patients, for long enough, to improve oral health.

## 10 Discussion and future perspectives

The three disease contexts considered in this review share general principles of attachment, spatial organization and community interaction, but the dominant ecological pressures differ. Cariogenic biofilms develop on a mineralized, non-living surface under repeated carbohydrate exposure, with acidogenic metabolism, acid tolerance and glucan-rich matrix contributing to localized demineralizing conditions (Kim et al., 2020; Cai and Kim, 2023). Periodontal biofilms occupy an inflamed subgingival habitat in which protein- and heme-rich host substrates, proteolytic metabolism and immune dysregulation reinforce one another (Hajishengallis et al., 2011; Hajishengallis and Lamont, 2021). Peri-implant biofilms assemble on a foreign material surface beneath a peri-implant mucosal interface and show marked subject-to-subject variation (Dieckow et al., 2024; Ghensi et al., 2025). A molecular determinant or therapeutic effect demonstrated in one of these settings should therefore not be assumed to transfer unchanged to the others.

The major challenge in oral biofilm research is no longer documenting microbial diversity. The field can identify community members at high taxonomic resolution, measure thousands of microbial transcripts and proteins, detect metabolites across oral sites, reconstruct metabolic networks and visualize selected taxa and physicochemical gradients at micrometre scales. What remains harder is determining which observations represent causal processes that can be altered safely and durably.

Future progress should therefore prioritize perturbation. A useful experimental sequence is: identify a reproducible community feature, formulate a mechanistic hypothesis, perturb the predicted determinant, determine whether spatial and metabolic consequences follow, and then test whether those effects persist as biological complexity is restored.

Defined communities are most useful when their members represent the functions required to test a specific hypothesis, rather than when they are chosen simply to maximize taxonomic breadth. A cariogenic model, for instance, might bring together organisms representing EPS production, acid generation, lactate utilization, peroxide-mediated antagonism and alkali production, while a periodontal model might combine early colonization, bridging interactions, proteolytic metabolism, heme utilization and host inflammatory signaling. Patient-derived microcosms should then serve as a second validation layer, one that keeps the donor variability that may ultimately determine therapeutic response (Fernandez Y Mostajo et al., 2017; Adami et al., 2025).

Longitudinal designs are equally important because attachment, matrix accumulation, metabolic compartmentalization and biofilm aging are biologically distinct states. Interventions should therefore be evaluated before exposure, during treatment and after withdrawal. The post-treatment phase is especially informative because it shows whether the community returns toward its original state, remains altered, or stabilizes in a different configuration.

Perturbation should remain central to functional-genomic studies. Candidate genes from Tn-seq or arrayed libraries should be reconstructed independently, complemented and, where appropriate, whole-genome verified before mechanistic assignment (Solano Morales et al., 2026). CRISPRi can extend this logic to essential genes, with graded repression distinguishing general fitness loss from a disproportionate effect on biofilm physiology (Shields et al., 2020; St. Pierre et al., 2024).

Spatial context must also be preserved. Micron-scale imaging shows non-random plaque architecture (Mark Welch et al., 2016; Kim et al., 2020), pH measurements reveal substantial local acidity gradients (Kristensen et al., 2023), and pH-O2 ratiometry shows that relationships between acidity and oxygen depend on community context (Lynge et al., 2026). Methods should be selected according to the scale of the proposed mechanism rather than technological novelty.

Host context deserves a place earlier in therapeutic development, not as an afterthought. In periodontitis, microbial and inflammatory processes reinforce each other (Hajishengallis et al., 2011; Maekawa et al., 2014; Hajishengallis and Lamont, 2021; Wang et al., 2022), and even simulated crevicular flow on its own can change microbial clearance and inflammatory output (Makkar et al., 2023). Caries interventions need disease-proximal endpoints for the same reason: enamel demineralization can improve without any measurable drop in total biofilm biomass (Marin et al., 2022).

Computational models are most valuable when they guide experimental design rather than substitute for it. Genome-scale models can nominate nutrient dependencies (Mazumdar et al., 2009; Bernstein et al., 2019; Jijakli and Jensen, 2019; Jensen et al., 2020; Pucci et al., 2026), networks can identify phenotype-associated restructuring (Espinoza et al., 2022), machine learning can prioritize microbial combinations associated with risk (Grier et al., 2021; Wei et al., 2025; Seo et al., 2026; Xie et al., 2026), and structure-based screening can select compounds for experimental follow-up (Zhang et al., 2017; De Lucena et al., 2026). These predictions become mechanistically informative only when they are converted into experimentally falsifiable hypotheses.

As defined in Section 1, precision can operate at organism, mechanistic, spatial, ecological or patient levels. The practical implication is that selectivity must be demonstrated at the level being claimed: target specificity does not guarantee ecological sparing, and patient stratification will require evidence that treatment response varies reproducibly with host or community context.

Ecological safety should be evaluated alongside efficacy. Relevant measurements include community diversity, health-associated taxa, metabolic function, local chemistry, matrix architecture, resilience after treatment withdrawal and host inflammatory responses. An intervention should be described as “microbiome preserving” only when preservation has been measured rather than inferred.

Therapeutic development also benefits from an explicit evidence hierarchy, moving from computational or biochemical activity through monospecies and defined multispecies systems, patient-derived communities, ex-vivo or animal validation, human proof-of-mechanism and, ultimately, controlled clinical outcomes. The order need not be rigid, but each stage addresses an evidentiary question that must be resolved somewhere in the development pathway.

## 11 Conclusion

Oral biofilms are not simply accumulations of bacteria attached to a surface. Their biological properties emerge from the spatial organization of microbial cells, receptor-mediated adhesion, extracellular matrix construction, metabolic exchange, local physicochemical gradients and continuous interaction with the host. These processes depend on one another, but none of them can substitute for another.

The extracellular matrix makes this kind of integration especially easy to see. Glucans provide structural material and additional binding surfaces, eDNA originates through several release mechanisms, surface-associated proteins can acquire extracellular functions, and membrane vesicles extend biologically active material beyond the producing cell. At the same time, metabolism generates local chemical environments that influence which organisms grow and how therapeutic agents perform.

The same evidentiary discipline applies throughout: candidate determinants identified by genetics, multi-omics or computational analysis require experimental resolution before they are treated as mechanism, and therapeutic evidence requires an equivalent hierarchy, in which a reduction in biomass or loss of a target species is not equivalent to ecological restoration or clinical benefit.

Future progress will therefore require a shift from describing oral biofilms toward perturbing them at the appropriate biological scale. The most informative studies will integrate microbial identity, spatial architecture, extracellular matrix, community metabolism and host environment, then test whether a defined intervention alters a disease-relevant function without producing unacceptable ecological or host consequences. Precision oral biofilm control is unlikely to depend on indiscriminate removal of microorganisms. A more realistic objective is to identify which molecular or ecological processes sustain a damaging community in a particular environment, intervene at those processes, and determine whether the resulting ecosystem is both stable and compatible with oral health.
